# Social Media Engagement and Influenza Vaccination During the COVID-19 Pandemic: Cross-sectional Survey Study

**DOI:** 10.2196/25977

**Published:** 2021-03-16

**Authors:** Arriel Benis, Anna Khodos, Sivan Ran, Eugene Levner, Shai Ashkenazi

**Affiliations:** 1 Faculty of Industrial Engineering and Technology Management Holon Institute of Technology Holon Israel; 2 Faculty of Digital Technologies in Medicine Holon Institute of Technology Holon Israel; 3 Faculty of Sciences Holon Institute of Technology Holon Israel; 4 Adelson School of Medicine Ariel University Ariel Israel

**Keywords:** influenza, vaccines, vaccination, social media, online social networking, health literacy, eHealth, information dissemination, access to information, COVID-19

## Abstract

**Background:**

Vaccines are one of the most important achievements of modern medicine. However, their acceptance is only partial, with vaccine hesitancy and refusal representing a major health threat. Influenza vaccines have low compliance since repeated, annual vaccination is required. Influenza vaccines stimulate discussions both in the real world and online. Social media is currently a significant source of health and medical information. Elucidating the association between social media engagement and influenza vaccination is important and may be applicable to other vaccines, including ones against COVID-19.

**Objective:**

The goal of this study is to characterize profiles of social media engagement regarding the influenza vaccine and their association with knowledge and compliance in order to support improvement of future web-associated vaccination campaigns.

**Methods:**

A weblink to an online survey in Hebrew was disseminated over social media and messaging platforms. The survey answers were collected during April 2020. Anonymous and volunteer participants aged 21 years and over answered 30 questions related to sociodemographics; social media usage; influenza- and vaccine-related knowledge and behavior; health-related information searching, its reliability, and its influence; and COVID-19-related information searching. A univariate descriptive data analysis was performed, followed by multivariate analysis via building a decision tree to define the most important attributes associated with vaccination compliance.

**Results:**

A total of 213 subjects responded to the survey, of whom 207 were included in the analysis; the majority of the respondents were female, were aged 21 to 40 years, had 1 to 2 children, lived in central Israel, were secular Israeli natives, had higher education, and had a salary close to the national average. Most respondents (128/207, 61.8%) were not vaccinated against influenza in 2019 and used social media. Participants that used social media were younger, secular, and living in high-density agglomerations and had lower influenza vaccination rates. The perceived influence and reliability of the information on social media about COVID-19 were generally similar to those perceptions about influenza.

**Conclusions:**

Using social media is negatively linked to compliance with seasonal influenza vaccination in this study. A high proportion of noncompliant individuals can lead to increased consumption of health care services and can, therefore, overload these health services. This is particularly crucial with a concomitant outbreak, such as COVID-19. Health care professionals should use improved and targeted health communication campaigns with the aid of experts in social media. Targeted communication, based on sociodemographic factors and personalized social media usage, might increase influenza vaccination rates and compliance with other vaccines as well.

## Introduction

### Background

Influenza is an acute, viral infectious disease characterized by high fever, cough, runny nose, muscle pain, joint pain, and severe exhaustion [1]. It is associated with multiple complications, including hepatitis, encephalitis, muscle tissue destruction, renal impairment, and secondary bacterial infection (ie, pneumonia, sinusitis, and, in children, middle ear infection) [2]. Although vaccines are one of the most important achievements of modern medicine, their acceptance among the population is only partial; this lack of compliance has been identified by the World Health Organization (WHO) as one of the major threats to public health. A wide variety of communication channels are now being used to improve worldwide vaccine responsiveness. There is a need to improve the efficacy of online communication, which is currently a significant source of health and medical information [3].

According to the WHO, every year around 1.5 billion people suffer from seasonal influenza, of whom 3 to 5 million have a serious illness, sometimes requiring hospitalization, and 650,000 die [1]. However, the percentage of the population who comply with seasonal vaccination against it is relatively low [4,5]. For example, in Israel, around 25% of the population are vaccinated against influenza each winter [6]. The second type of influenza relates to major changes in viral antigens occurring every few decades and, thus, leads to new influenza strains that are unfamiliar to the human immune system, causing a worldwide epidemic, called a pandemic, with tens of millions of sick patients and millions of deaths [1]. This occurred with the 1918 influenza pandemic, in which about one-third of the world’s population was ill and 50 to 100 million people died [7-9].

Over the last decades, the internet has supported the monitoring, prediction, and surveillance (ie, infoveillance [10-12]) of epidemics and the behavior of the population, specifically in the context of influenza [13-15]. Furthermore, valuable information is discoverable, like early warnings of disease outbreaks, dissemination tracking, and resilience [16]. Social media and social networking services (SNSs) are powerful internet-based communication tools [17,18]. Each SNS has a variety of functionalities and goals. Facebook is a grand public-focused polyvalent platform. LinkedIn focuses on professional networking. Instagram, Flickr, and Pinterest deal with video and picture sharing. Twitter and Tumblr allow for blogging and microblogging. Reddit provides news aggregation, web content rating, and blogging services. Telegram is primarily used for instant and broadcast messaging to an unlimited number of subscribers over dedicated channels. Social media and SNSs, in particular, are also well known for disseminating evidence-based health care information and recommendations [14]. However, efficient and effective health-related information must be monitored and controlled for both *quality and reliability* [19], and *confidentiality, privacy, and ethics* of contacts between health care information customers and providers [20]. The social media impact must be understood in the field of health communication [21,22]. Accordingly, the development of relevant policies is needed [23,24] for reducing the risk and impacts of the *misinformation epidemic* or the *infodemic* spread on media, and for building the appropriate capacities to support eHealth and science literacy [25,26]. There is a crucial need for public health decision makers who are concerned with disease literacy to give the health information providers appropriate tools for efficiently disseminating the information, taking into account possible personal and environmental influences [12].

Vaccination against influenza is a significant and cost-effective protective mechanism for reducing the disease burden related to its morbidity and mortality [5]. Nevertheless, at the population level, its coverage is insufficient due to factors influencing vaccination decisions and hesitancy, such as risk-benefit misperception or accessibility to the health care system [27]. A major contribution to these factors is communication, involving both social and mass media, family, friends, and health care professionals [28]. More specifically, social media could affect the compliance of the population to vaccination guidelines [27]. For example, those who advocate against vaccines use social media to disseminate their messages on a large scale, increasing vaccine hesitancy or refusal in the population [29].

Concerning the new COVID-19 vaccines, evaluating the relationship between the population’s perception of and compliance with the vaccine against influenza is important. Therefore, this will contribute to creating effective means of online communication to improve vaccine acceptance [30-33].

### Aims and Objectives

Social media and SNSs have been used to improve vaccine response worldwide. However, they are also a forum for vaccine opponents and spreading of fake news. Understanding social media engagement, influence, and reliability is a critical point for improving the efficacy of advertising and publicity policies on social media. Our primary aim is to support the design and the implementation of future eHealth strategies and interventions on social media to increase the quality of targeted communication campaigns and the influenza vaccination rates [6,34-36]. Our main objective is to describe and characterize profiles regarding influenza vaccination and their association with social media engagement, influence, and reliability. We specifically focus on the Israeli population in this study. The findings of this research may then support vaccination campaigns against COVID-19.

This cross-sectional survey-based research is led by three hypotheses:

1. The use of social media influences the vaccination compliance of health care customers.
2. Influenza vaccination compliance is affected by social factors and by perceptions, reliability, and influence of information from social media.
3. The perceived influence and reliability of information from social media about SARS-CoV-2 or COVID-19 is similar to that regarding influenza.

Our goal is to identify sociodemographic and social media engagement attributes affecting influenza vaccination compliance. This research characterizes the differences between individuals vaccinated or not vaccinated against influenza during the 2019 season [37]. We attempt to understand whether there is a link between seasonal vaccination against influenza and social media engagement, influence, and perception of reliability during the COVID-19 pandemic.

This survey was granted ethical approval by the Ethics Committee of the Faculty of Technology Management of the Holon Institute of Technology (TM/2/2020/AB/002). The information provided by the participants during the survey are stored in a secured, encrypted manner, with restricted access provided by the institution of the principal researcher (AB).

## Methods

### Overview

We performed a cross-sectional survey of volunteers and anonymous Hebrew speakers over the internet about their online social network habits and their behavior concerning influenza vaccination. The survey was conducted over 14 days, between April 14 and 28, 2020, coinciding with the end of the seasonal influenza outbreak as well as the second month of the COVID-19 pandemic in Israel. Israel is a country in which a high percentage of the population uses the internet. With more than 80% of the population having at least one account on an SNS [38], it is among the highest in the world and is continuously increasing. Facebook is the leading social media platform used in Israel and the percentage of its users is continuously growing (eg, in April 2020: 62.87%; in August 2020: 86.47%) [39].

Upon consent, 213 participants were instructed to complete a 30-question survey asking about their usage and perception of health information available on social media, its reliability, and its influence on their compliance to vaccinate against influenza (see Multimedia Appendix 1) [40,41].

The survey was hosted on an Israeli website for the management of surveys in Hebrew—the IMKFORMS system—and its address was disseminated by publishing it on SNSs (ie, Facebook, Twitter, and LinkedIn) and instant messaging platforms (ie, WhatsApp and Telegram) and by sending its link via email to personal and professional contact lists.

The questionnaire included five subsets of questions, each with a specific focus:

1. Sociodemographics (9 questions), including gender, age range, relationship status, number of children, area of residence, country of birth, religious affiliation, education, and monthly income.
2. Social media usage (2 questions), including the self-estimated daily amount of social media use and the types of involvement on different social media platforms.
3. Influenza and vaccine-related knowledge and behavior (5 questions), including vaccine status in 2019, knowledge about the influenza vaccine, reasons for taking the seasonal influenza vaccine, and chronic disease in the family.
4. Health-related information searching and publishing, its reliability, and its influence (9 questions), including confidence in sources of information and searching for information, reliability and influence, and types and intensity of involvement on different social media platforms regarding health, vaccines, and influenza.
5. COVID-19- and vaccine-related information searching and publishing, its reliability, and its influence (5 questions), including sources and searching for information, reliability and influence, and types and intensity of involvement on different social media platforms regarding the COVID-19 pandemic.

The questions dealing with social media usage, reliability, and influence were in the form of matrix point rating multiple-choice questions with 4-point Likert scales.

### Data Analysis

By using the exclusion criteria, we removed the full answer sets of responders who declared residence outside Israel or who did not answer at least one of the sociodemographic questions. We redefined some categories to facilitate the data analysis by working with groups comprising, as much as possible, the largest number of similar answers (eg, age groups, relationship status, number of children, area of residence, country of birth, education, and social media activity, reliability, and influence). Descriptive statistics such as frequencies and proportions were computed. Chi-square tests and Fisher exact tests were used to compare categorical variables. Cronbach α was used to measure the internal reliability of social media usage, reliability, and influence (Cronbach α=.949). The categorical variables were presented as numbers and percentages. Statistical significance was considered with a 2-sided *P* value of .05 or less. However, the borderline values have not been considered as not being significant in the evaluation [42]. To promote the effective focusing of communication to encourage vaccination against influenza, a set of factors were considered. Building a decision tree allowed, in this case, the definition of target profiles and the overcoming of Simpson's paradox, which may limit the quality of the decision support provided to decision makers. This phenomenon describes situations in which a trend appears in some groups of data but disappears when these groups are aggregated and vice versa [43]. Therefore, these learning classifiers allow nonlinear interactions between attributes and are easily interpretable. The decision tree for predicting vaccinated and unvaccinated profiles [44] was built by considering attributes with *P*≤.10 that were used in further multivariate analysis.

The data analysis was performed with R, version 4.0.2 (The R Foundation). The *psych* package [45] was used for computing the internal consistency of reliability of the answers to the matrix multipoint scale questions. The *compareGroups* package [46] was used for statistical computations. The *rpart* package [47] was used for the decision tree processing [48].

The manuscript adheres to reporting standards, including the Checklist for Reporting Results of Internet E-Surveys (CHERRIES) and the Strengthening the Reporting of Observational Studies in Epidemiology (STROBE) guidelines [49-51].

## Results

### Study Population

#### Overview

The population of survey participants (see Table 1) includes 207 individuals after applying the exclusion criteria on 213 total responders. A substantial proportion of the participants were female (126/207, 60.9%), between the ages of 21 and 40 years (47/207, 71.0%), in a relationship (156/207, 75.4%), with 1 to 2 children (101/207, 48.8%), and living in central Israel (130/207, 62.8%). Additionally, the majority of survey participants were Israeli natives (116/207, 56.0%), had a secular affiliation (144/207, 69.6%), possessed higher education (182/207, 87.9%), and had a salary near the national average (81/207, 39.1%), which is around 12,000 New Israeli Shekels per month (US $3092.78) [52]. Regarding demography of social media usage and vaccination status, the majority of respondents were not vaccinated against influenza in 2019 (128/207, 61.8%) and used social media (119/207, 57.5%).

**Table 1:** Sociodemographic characteristics of the survey population.

Characteristic		All participants (N=207), n (%)	Social media user			Vaccinated in 2019		
			Yes (n=119), n (%)	No (n=88), n (%)	*P* value	Yes (n=79), n (%)	No (n=128), n (%)	*P* value
**Vaccinated in 2019**					<.001			N/A^a^
	Yes	79 (38.2)	31 (26.1)	48 (55)	—^b^	N/A	N/A	N/A
	No	128 (61.8)	88 (73.9)	40 (45)	—	N/A	N/A	N/A
**Social media user**					N/A			<.001
	Yes	119 (57.5)	N/A	N/A	N/A	31 (39)	88 (68.8)	—
	No	88 (42.5)	N/A	N/A	N/A	48 (61)	40 (31.2)	—
**Gender**					.07			.11
	Male	81 (39.1)	53 (44.5)	28 (32)	—	25 (32)	56 (43.8)	—
	Female	126 (60.9)	66 (55.5)	60 (68)	—	54 (68)	72 (56.2)	—
**Age category (years)**					.002			.11
	21-30	48 (23.2)	34 (28.6)	14 (16)	—	12 (15)	36 (28.1)	—
	31-40	99 (47.8)	63 (52.9)	36 (41)	—	41 (52)	58 (45.3)	—
	41-50	38 (18.4)	14 (11.8)	24 (27)	—	15 (19)	23 (18.0)	—
	51-60	15 (7.2)	7 (5.9)	8 (9)	—	6 (8)	9 (7.0)	—
	≥61	7 (3.4)	1 (0.8)	6 (7)	—	5 (6)	2 (1.6)	—
**Relationship status**					.04			.008
	Not in a relationship	51 (24.6)	36 (30.3)	15 (17)	—	11 (14)	40 (31.2)	—
	In a relationship	156 (75.4)	83 (69.7)	73 (83)	—	68 (86)	88 (68.8)	—
**No. of children**					.009			.03
	0	58 (28.0)	43 (36.1)	15 (17)	—	14 (18)	44 (34.4)	—
	1-2	101 (48.8)	53 (44.5)	48 (55)	—	45 (57)	56 (43.8)	—
	3-6	48 (23.2)	23 (19.3)	25 (28)	—	20 (25)	28 (21.9)	—
**Residence**					.02			.53
	Center	130 (62.8)	66 (55.5)	64 (73)	—	47 (59)	83 (64.8)	—
	Periphery	77 (37.2)	53 (44.5)	24 (27)	—	32 (41)	45 (35.2)	—
**Country of birth**					.002			.82
	Israel	116 (56.0)	55 (46.2)	61 (69)	—	43 (54)	73 (57.0)	—
	Aboard	91 (44.0)	64 (53.8)	27 (31)	—	36 (46)	55 (43.0)	—
**Religious affiliation**					.21			.03
	Secular	144 (69.6)	89 (74.8)	55 (63)	—	50 (63)	94 (73.4)	—
	Traditional	40 (19.3)	19 (16.0)	21 (24)	—	15 (19)	25 (19.5)	—
	Religious	17 (8.2)	7 (5.9)	10 (11)	—	12 (15)	5 (3.9)	—
	Other	6 (2.9)	4 (3.4)	2 (2)	—	2 (3)	4 (3.1)	—
**Education (years)**					.71			.99
	≤12	25 (12.1)	13 (10.9)	12 (14)	—	9 (11)	16 (12.5)	—
	>12	182 (87.9)	106 (89.1)	76 (86)	—	70 (89)	112 (87.5)	—
**Monthly gross income (NIS^c^)**					.28			.75
	5001-10,000	33 (15.9)	24 (20.2)	9 (10)	—	10 (13)	23 (18.0)	—
	10,001-20,000	81 (39.1)	42 (35.3)	39 (44)	—	35 (44)	46 (35.9)	—
	20,001-25,000	28 (13.5)	16 (13.4)	12 (14)	—	9 (11)	19 (14.8)	—
	25,001-30,000	20 (9.7)	14 (11.8)	6 (7)	—	7 (9)	13 (10.2)	—
	≥30,001	25 (12.1)	13 (10.9)	12 (14)	—	9 (11)	16 (12.5)	—
	Did not disclose	20 (9.7)	10 (8.4)	10 (11)	—	9 (11)	11 (8.6)	—
**Do you or a family member^d^ have a chronic disease?**					.90			.45
	Yes	94 (45.4)	55 (46.2)	39 (44)	—	39 (49)	55 (43.0)	—
	No	113 (54.6)	64 (53.8)	49 (56)	—	40 (51)	73 (57.0)	—

^a^N/A: not applicable; irrelevant test.

^b^*P* values were calculated for categories and not for individual subcategories.

^c^NIS: New Israeli Shekel; the currency exchange rate at the time of publication was US $1 for NIS 3.88.

^d^Up to a second degree.

#### Social Media Users

There were significantly fewer social media users vaccinated against influenza in 2019 (31/109, 26.1%; *P*<.001) compared to nonusers (48/88, 55%). In both groups, the responders were predominantly female (users: 66/119, 55.5% vs nonusers: 60/88, 68%; *P*=.09) and in a relationship (83/119, 69.7% vs 73/88, 83%; *P*=.04). However, the social media users were globally younger, 21 to 40 years old (97/119, 81.5% vs 50/88, 67%), whereas the nonusers were older, aged between 31 and 60 years (68/88, 77%). Similarly, the former group often had no children (36/118, 80.6%), whereas the nonusers had children in most cases (73/88, 83%). Still, most respondents lived in central Israel (66/119, 55.5% and 64/88, 73%), but residents of the periphery used significantly more social media than those living in the center (53/119, 44.5% vs 24/88, 27%; *P*=.02). Additionally, in the survey population, Israeli natives used social media significantly less than immigrants (55/119, 46.2% vs 64/119, 53.8%; *P*=.002).

Furthermore, the proportion of people of traditional or religious affiliation who did not use social media was higher than their proportion in the group of users (31/88, 35% vs 26/119, 21.8%; *P*=.21). The level of education, declared monthly gross income, and experience of chronic disease by themselves or by a family member did not appear to be a determinant of overall social media use (*P*=.28, *P*=.71, and *P*=.90, respectively).

#### Vaccinated Versus Unvaccinated in 2019

The percentage of respondents vaccinated against influenza in 2019 was significantly (*P*<.001) higher in the group of social media nonusers than in the users’ group (48/79, 61% vs 31/79, 39%); inversely, the percentage of unvaccinated respondents was higher in the group of social media users (88/128, 68.8% vs 40/128, 31.2%). Furthermore, the proportion of vaccinated people was higher when the responders were in a relationship (68/79, 86% vs 88/128, 68.8%; *P*=.008) and had children (65/79, 83% vs 84/128, 65.7%; *P*=.03). The degree of religious affiliation provided critical insight into this study. The secular participants in the survey represented a higher proportion of unvaccinated people (unvaccinated: 94/128, 73.4% vs vaccinated: 50/79, 63%), while those with religious affiliations were more compliant (unvaccinated: 5/128, 3.9% vs vaccinated: 12/79, 15%). Similar to the use of social media, influenza vaccination was not associated with chronic disease, personally or among family members (*P*=.45).

### Social Media Usage and Vaccination Status

**Figure 1:**
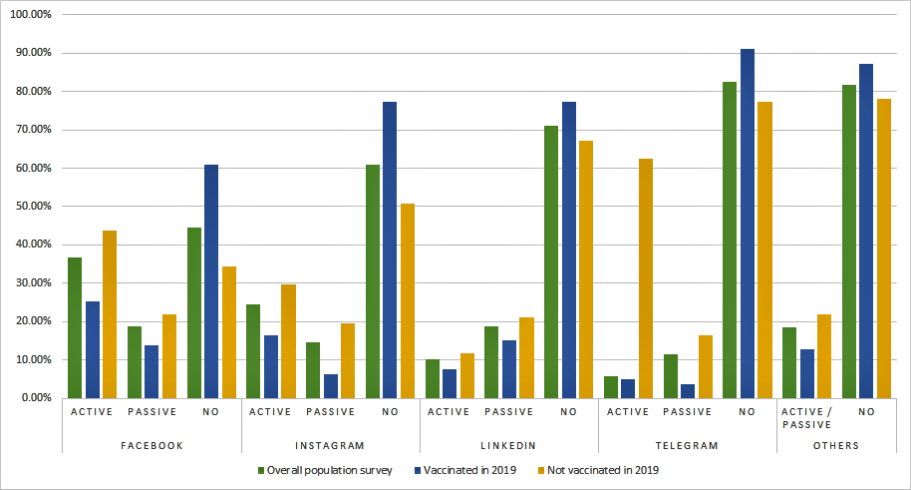
Social media usage and vaccination status against influenza in 2019.

The participants in the survey were asked to specify which social media platforms they used actively (ie, publishing or reacting to posts), passively (ie, reading posts), or not at all (see Figure 1 and Multimedia Appendix 2). The participants declared using Facebook (115/207, 55.0%), Instagram (81/207, 39.1%), LinkedIn (60/207, 28.9%), Telegram (36/207, 17.4%), and others (ie, Twitter, Tumblr, Reddit, Flickr, and other social media platforms) as a whole (38/207, 18.4%). More accurately, Facebook, Instagram, and Telegram users were significantly (*P*=.001) less vaccinated in 2019. The use of LinkedIn or other unspecified social media platforms was not significantly associated with vaccination status (*P*=.30 and *P*=.14, respectively).

Moreover, no significant difference was observed between the groups regarding the belief that the vaccine against influenza caused the disease (between 62.0% and 68.0%; *P*>.47), nonetheless a majority of participants knew that the influenza vaccine is an attenuated or inactivated virus (150/207, 72.5%).

### Reasons for Receiving the Influenza Vaccine in 2019

**Figure 2:**
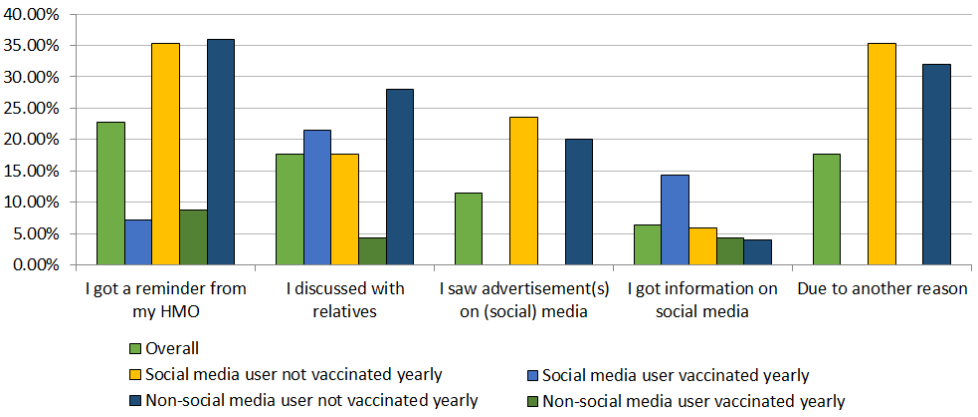
Reasons for receiving the influenza vaccine in 2019. The plot shows all participants and stratification by social media use and vaccination against influenza in 2019. HMO: health maintenance organization.

The reasons for receiving the annual influenza vaccine varied from one individual to another (see Figure 2 and Multimedia Appendix 3). The participants vaccinated in 2019 (79/207, 38.2%) were divided into *social media users* (31/79, 39%) and *nonusers of social media* (48/79, 61%). The proportion of yearly vaccinated individuals was similar in both groups (14/31, 45% vs 23/48, 48%). Receiving a reminder from a health maintenance organization (HMO) seemed to significantly influence the compliance with vaccination for the group of individuals who were not vaccinated annually (6/7, 86%; *P*=.09 vs 9/11, 82%; *P*=.06). Among those who have discussed the issue with relatives, seen advertisements in the media, or gotten information via social media, some differences were noticeable. The social media users seeing advertisements may have been influenced by these communication tools and took the vaccine in 2019 in contrast to previous years (4/9, 44%). A relatively high proportion (14/79, 18%) of individuals were vaccinated in 2019 but had not been vaccinated annually. This may be associated with the COVID-19 pandemic and the worry that it induced [53]. Moreover, a majority of the responders who were not vaccinated yearly received the vaccine after a discussion with relatives when they were not social media users (7/8, 88% vs 3/6, 50%; odds ratio 7.0, 95% CI 0.50-97.75; *P*=.15).

### Searching for and Publishing Information Related to Health, Specifically to Influenza Vaccines

Social media is the main source of information and news consumption [54,55]. Anyone can post content and thus publish “information” (see Multimedia Appendix 4). No significant differences were observed in health information–related searches, influenza vaccine–related posting, and COVID-19-related posting (*P*>.30). However, declared behavior was significantly associated with vaccination against influenza and with the search for influenza vaccine– and COVID-19-related information (22/79, 28% vs 11/128, 8.6%; *P*<.001, and 26/79, 33% vs 23/128, 18.0%; *P*=.02, respectively), as well as publishing of health-related information (46/79, 58% vs 56/128, 43.8%; *P*=.06). These results show that vaccinated individuals were more active and involved in their health management by searching for and sharing relevant information.

### Reliability, Influence, and Confidence Perceptions of Influenza-, Vaccine-, and COVID-19-Related Information on Social Media Platforms

The survey participants were asked to report their perceptions of reliability, influence, and confidence of influenza-, vaccine-, and COVID-19-related information available on the social media platforms (see Multimedia Appendices 5 and 6). The proportion of participants with *no opinion* was relatively high (at least 118/207, 57.0%) and higher than that in the *social media nonusers* group (88/207, 42.5%). The *no opinion* answer was not considered as a full lack of positioning but rather as a lack of use, knowledge, or understanding of a platform. It seems that the social media users were not aware of the impacts of these platforms on their behaviors. Facebook received the highest score of reliability (71/207, 34.3%; *P*=.06 and 74/207, 35.7%; *P*=.003) and the score was higher in the *nonvaccinated* group (50/128, 39.1%). The influence of the information about the influenza vaccine was not considered as being substantial (53/207, 25.6%). For COVID-19, the results were different: two-thirds of the participants, not vaccinated and having an opinion, were influenced by the information appearing on social media.

The users’ trust in the source of information is crucial. The majority of participants had confidence in *governmental and health organizations* (overall: 155/207, 74.9%; vaccinated: 65/79, 82%; nonvaccinated: 38/128, 29.7%). Interestingly, the participants generally had less confidence in health care professionals as a source of influenza vaccine–related information (overall: 84/207, 40.6%; vaccinated: 39/79, 49%; nonvaccinated: 45/128, 35.2%; *P*=.06). These two sources represent those with the highest levels of trust and, as such, the highest levels of influence. One-third of the participants were confident in scientific publications (70/207, 33.8%). Moreover, the information provided by relatives had some credibility (17/207, 8.2%). The pharmaceutical industry and the vaccine opponents’ information were strongly rejected (>97.6%).

### Multivariate Analysis

The multivariate analysis consisted of building decision trees enabling the classification of individuals to be vaccinated or unvaccinated against influenza and so defining target profiles for increasing vaccination engagement. As presented in the univariate analysis (Multimedia Appendices 2-7), multiple decision trees were built with the subsets of attributes (see Multimedia Appendix 8) without considering the *P* values, with and without using the COVID-19-related set. The decision trees were built using a training data set of 66.2% (137/207) randomly selected records and their prediction capabilities were tested on the rest (70/207, 33.8%).

One decision tree was built with all attributes with *P*≤.10 and with the exclusion of the COVID-19-related attributes (56/70, 80% of the test samples were rightly classified as vaccinated or not). The root of this decision tree was *due to another reason* as the reason given for being vaccinated in the 2019 season. In the overall survey, this represented 18% (14/79) of the vaccinated individuals or 6.8% of the responders (14/207). Moreover, the same group reported that they were not vaccinated annually (see Multimedia Appendix 3). To build a decision tree that supports efficient decision making by the domain expert, this attribute did not allow the targeting of a specific subpopulation. Hence, the increase in the number of people vaccinated may be due to fear of the pandemic [53] and the popular misunderstanding of the differences between seasonal influenza and SARS-CoV-2 (ie, the COVID-19 pandemic) [33].

**Figure 3:**
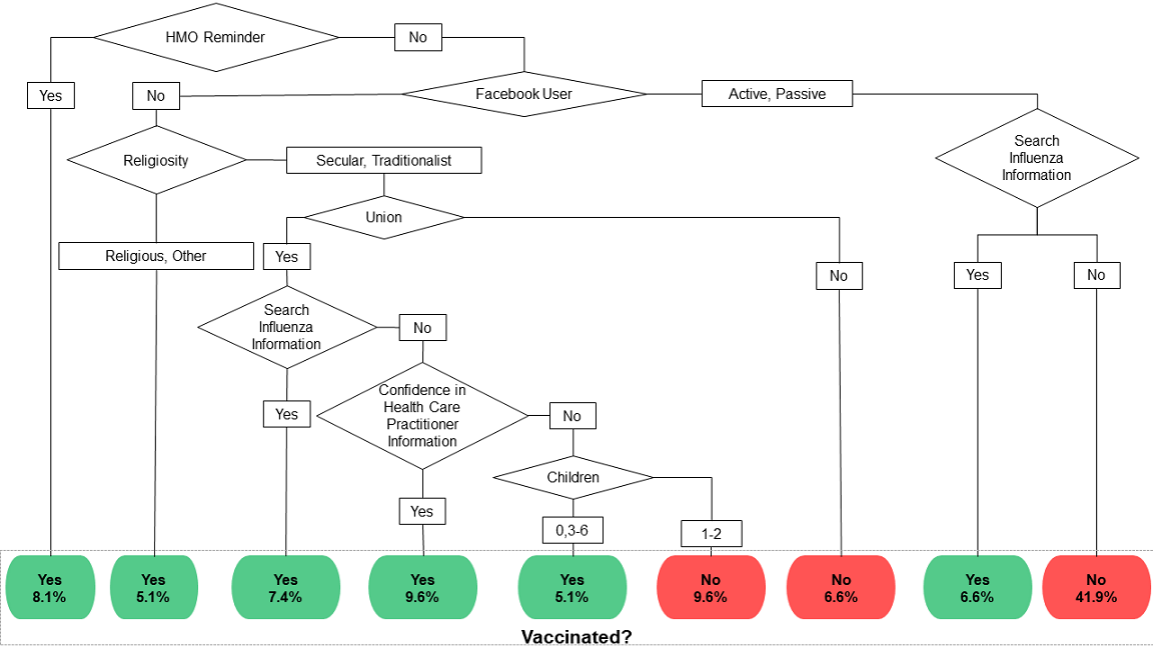
Decision tree predicting vaccinated individuals based on all survey attributes with *P*≤.10 and without the *due to another reason* attribute. HMO: health maintenance organization.

Consequently, two decision trees were built without the *due to another reason* attribute: the first was built on all attributes with *P*≤.10, and the second further excluded the COVID-19-related attributes as inputs. The same decision tree was generated (see Figure 3), with an overall performance of 76% (53/70 of the test sample were rightly classified as vaccinated or not). The difference in the classification performances of the trees with the declaration *due to another reason* or without it was 4% (3/70). This means that the majority (11/14, 79%) of the individuals vaccinated for this *reason* were rightly classified even without taking it into account.

Not focusing particularly on COVID-19 did not have an impact on classification. This supports our main objective, which was to characterize profiles of vaccination engagement in a simple and generalizable way.

The root of the proposed decision tree (see Figure 3) was a reason for obtaining the seasonal vaccine and it was a nonsocial attribute: *I got a reminder from my HMO* (11/137, 8.1% of the training set; *P*=.08). This reminder was sent by HMOs via SMS and the majority of those who received it were compliant and vaccinated (see Figure 3 and Multimedia Appendix 4).

The next node involved discrimination between Facebook users and nonusers (node *Facebook User*). The group of individuals engaged with this social media platform were less vaccinated than its nonusers. However, they actively searched for information related to influenza vaccines (ie, *Search Influenza Information*); thus, their health literacy seemed to positively influence vaccination compliance (9/70, 7%; *P*=.07).

On the other hand, the Facebook nonusers were then split by their religious practice (ie, *Religiosity*). The individuals who were not vaccinated because they did not receive an HMO reminder and were also not users of Facebook were, for the majority, vaccinated if they were *religious* or had another level of practice, meaning neither *secular* nor *traditional* (7/137, 5.1%; *P*=.05). In the last node, those not in a relationship were more likely not to be vaccinated (9/137, 6.6%; *P*=.07).

The majority of those who identified as secular or traditional, were in a relationship, and searched for influenza vaccine information were also vaccinated (10/137, 7.4%; *P*=.07). Those who did not search for information but were confident in the information provided by health care practitioners also showed good compliance (13/137, 9.6%; *P*=.10). Surprisingly, those who were not confident, and who had no children or more than 3 children, were often vaccinated (7/137, 5.1%; *P*=.05), while parents of 1 or 2 children were less often vaccinated (13/137, 9.6%; *P*=.10).

## Discussion

### Principal Findings

This study aimed to quantify the contribution of social media and its perceived reliability to modern health care customer behavior, thus highlighting the centrality of these media platforms to influence treatment and, specifically, compliance with influenza vaccination [56,57]. The most important outcome of this study is building a decision tree that is based on our findings and supports a multivariate and integrative viewpoint. It is actually reasonable that various interacting links exist between the seasonal vaccination against influenza and social media engagement, influence, and reliability in Israel during the COVID-19 pandemic [39]. Indeed, being or not being a user of social media or searching for information about the influenza vaccine present strong associations with vaccination status. Nevertheless, the vaccination reminders sent by the HMO are one of the most crucial factors in vaccination compliance.

The results show that the use of social media influences vaccination compliance in the Israeli population. This is correlated with sociodemographic factors and with perceptions of influence and reliability of information from social media. Indeed, social media users were less frequently vaccinated than nonusers. More accurately, this group was composed of younger people with secular affiliations living in the center of the country (ie, high-density agglomeration) and were less vaccinated.

According to the data collected during the survey, social media is largely used in Israel by young (ie, <40 years old), urban (ie, specifically, living in densely populated central Israel), and more highly educated people. This research highlights that social media users and the majority of the nonvaccinated population are the younger population who also acknowledge their confidence in the reliability and influence of social media, concurrent with their low confidence in the information disseminated by governmental and health organizations and health professionals.

The perceived influence and reliability of the information on social media about COVID-19 are similar, in general, to those perceptions of the information about influenza. Accordingly, the risk of nonadherence to recommendations by governmental and health organizations to reduce the spread of these pandemic viruses (ie, hygiene measures, distancing, and vaccination) may be similar. Notably, while both parameters are relatively significant, the nonvaccinated subpopulation searches for information regarding influenza vaccination substantially less than information regarding SARS-CoV-2 or COVID-19. Social media platforms were used during the survey period by local, national, and international agencies (ie, governmental and health care organizations) to provide up-to-date facts, guidance, and directives to the public [58]. With this in mind, and considering the 2019-2020 influenza season time frame [37], 6.8% (14/207) of the respondents sought out the influenza vaccine, deviating from their habits.

### Strengths and Limitations

A major limitation of this study is that it was based on an online survey written in Hebrew and disseminated over the internet via social media, primarily to Israeli residents. This method limited the type of individuals who responded to the survey and the generalization of the results. Moreover, even though about half of the survey responders were not Israeli natives, the Hebrew language of the survey rendered it inaccessible to nonfluent Hebrew speakers (eg, new immigrants, residents who have not learned the language, and non-Hebrew speakers of the Arab sector). Furthermore, as the survey was disseminated over the internet, particularly by publication on social networks, it did not include representation of the ultra-Orthodox sector, which represents around 12% of the Israeli population. It is, therefore, suggested that similar studies be performed in other locations and using diverse languages. Still, Israel is recognized as one of the countries with high, and continuously increasing, penetration of internet and social media [38,39].

Regarding the strengths of the study, the seasonal influenza outbreaks and pandemics with the need for repeated vaccinations are an excellent model for understanding the behavior of the population, its risk perception [59], its fear [60], and the consequences of information fatigue [61]. Social media supports the dissemination of a constant flow of information from numerous sources. In the context of influenza and COVID-19, these sources are institutional and professional news channels, in parallel with mass populations who can easily share their opinions and information over any social media platform. Crisis-related communication uses social media to “communicate, self-organize, manage, and mitigate risks” and “make sense of the event” [62] in a rapid manner and on influential channels [63].

Indeed, social media and the internet, in general, are defined as common sources of information on measles and its vaccine. Similar to the findings of this study, use of social media is also associated with erroneous knowledge and noncompliance with vaccination recommendations against measles [64].

Health communication countermeasures must be developed to increase the efficiency of campaigns for vaccination [64]. They must be dynamically adapted over all the communication channels in order to increase engagement for influenza and other vaccines, thereby reducing the potential additional overload on health care organizations [65,66].

### Conclusions

Social media is currently a leading and user-centered source of health information. The information available about influenza and the vaccines against it appears reliable and influences its readers, and this is significantly correlated with seasonal influenza vaccination compliance. Accordingly, it is crucial to improve the targeting of health communication campaigns in social media in order to increase compliance.

A high proportion of noncompliant and, therefore, nonvaccinated individuals can lead to increased consumption of health care services and an overload of the system. Therefore, in the COVID-19 era, and similarly to other epidemics and pandemics, health care services are overwhelmed by an excess of sick patients. Consequently, efficient communication actions with the individual, familial, and societal spheres; education on the benefits of vaccination; and education on the risks associated with infection are essential.

A future intervention must be efficiently implemented by developing new health communication processes, considering vaccinated and nonvaccinated individual profiles defined in a periodically updated way by building, for example, specific decision trees as proposed in this research (see Figure 3). This must be based on improving the health communication flow on social media, in near-real time, by monitoring and adapting targeted campaigns for facing rapid information changes in the internet sphere (eg, breaking news and fake news) [67].

Furthermore, most of the participants in this research were *young*. Nowadays, social media and social networks are used across the population regardless of, for example, age, gender, relationship status, education, religious affiliation, and country of residence. Consequently, social media–based health communication aiming to increase treatment compliance and, more accurately herein, influenza vaccination must take into account sociodemographic variables at a local level for efficiently and effectively targeting individuals to be motivated to vaccinate. This means delivering information to social media users in language that is easy to understand, in the languages spoken by various native and immigrant communities in the country, to ensure that the information reaches all residents. Additionally, the use of reminders, similar to those on SMS, should be generalized to social media by utilizing tailored advertisements [68,69].

## Appendix

Multimedia Appendix 1 Questionnaire on the behavior of social media users regarding health and vaccine issues.

Multimedia Appendix 2 Social media general usage and vaccination status against influenza in 2019.

Multimedia Appendix 3 Reasons for obtaining the influenza vaccine in 2019. The plot shows all participants and stratification by social media use and vaccination against influenza in 2019.

Multimedia Appendix 4 Searching for and publishing information related to health and specifically to influenza vaccines and COVID-19 in the 12 months before participating in the survey.

Multimedia Appendix 5 Perception of reliability and influence of the information related to influenza and vaccine and COVID-19 available on the most used social media platforms.

Multimedia Appendix 6 Perception of reliability and influence of the information related to influenza and vaccine and COVID-19 available on the most used social media platforms between social media users and social media nonusers.

Multimedia Appendix 7 Confidence in sources of information about the vaccine against influenza.

Multimedia Appendix 8 Prediction capabilities of decision trees based on the survey data.

## Abbreviations

CHERRIES: Checklist for Reporting Results of Internet E-Surveys
HMO: health maintenance organization
SNS: social networking service
STROBE: Strengthening the Reporting of Observational Studies in Epidemiology
WHO: World Health Organization
