# Supplementary material for: Social Media Engagement and Influenza Vaccination During the COVID-19 Pandemic: Cross-sectional Survey Study
Source: J Med Internet Res. 2021 Mar 16;23(3):e25977. doi: 10.2196/25977 (PMC7968480; doi:10.2196/25977)
Supplement: Multimedia Appendix 1 [file jmir_v23i3e25977_app1.pdf]

Multimedia Appendix 1. Questionnaire

Questionnaire on the behavior of social media users regarding health and vaccines issues

Dear Potential Participant,
Information about the annual influenza epidemic and its vaccine is available all around social media. We would like to study the use, the confidence as reliability, and the influence of social media on compliance with the annual vaccination against influenza. In the current COVID-19 pandemic, this study will also allow us to investigate links between the perceived reliability and influence of the information available both about influenza, its vaccine, and COVID-19.
We request your agreement to complete this short questionnaire on the subject. Your agreement will consist in responding and submitting the questionnaire below. You may stop answering it at any stage.
We emphasize that this research will use only the responses to the questionnaire; none of your personal information.
Thanks for your contribution.
----

1. What is your gender?
   - 1.1. Male
   - 1.2. Female
   - 1.3. Not relevant
   - 1.4. Not interested to answer

2. What age range do you belong to?
   - 2.1. 12-17
   - 2.2. 18-20
   - 2.3. 21-30
   - 2.4. 31-40
   - 2.5. 41-50
   - 2.6. 51-60
   - 2.7. 61-70
   - 2.8. 71-80
   - 2.9. 80 and more
   - 2.10. Not interested to answer

3. What is your family status?
   - 3.1. Single
   - 3.2. Married / In a union
   - 3.3. Separated / Divorced
   - 3.4. Widower
   - 3.5. Not interested to answer

4. How many kids do you have?
   - 4.1. No children
   - 4.2. 1-2
   - 4.3. 3-5
   - 4.4. 6 or more
   - 4.5. Not interested in answering

5. What is your residential district?
   - 5.1. North
   - 5.2. Haifa
   - 5.3. Tel-Aviv
   - 5.4. Center
   - 5.5. Jerusalem
   - 5.6. Shfela
   - 5.7. South
   - 5.8. Judea and Samaria
   - 5.9. Outside of Israel
   - 5.10. Not interested in answering

6. What is your country/part of the world of origin?
    6.1. Israel
    6.2. U.S.A.
    6.3. Europe
    6.4. Arab countries
    6.5. Ethiopia
    6.6. Commonwealth
    6.7. Other
    6.8. Not interested in answering

7. Do you consider yourself
    7.1. Secular
    7.2. Traditionalist
    7.3. Religious
    7.4. Other
    7.5. Not interested in answering

8. What is your level of education?
    8.1. Without education
    8.2. Elementary education
    8.3. Partial high school education
    8.4. Full high school education
    8.5. Academic education
    8.6. Not interested in answering

9. In a routine period, what is the (gross) income level in your household?
    9.1. NIS 5,000 and less
    9.2. NIS 5,000 - NIS 10,000
    9.3. NIS 10,001 - NIS 15,000
    9.4. NIS 15,001 - NIS 20,000
    9.5. NIS 20,001 - NIS 25,000
    9.6. NIS 25,001 - NIS 30,000
    9.7. NIS 30,001 and higher
    9.8. Not interested in answering

10. In which social media are you active?

	Writes or initiates posts	Responds to posts	Only reads messages	Not relevant or not familiar or not user
Twitter				
Facebook				
Tumblr				
Telegram				
Instagram				
Reddit				
Flickr				

Pinterest				
LinkedIn				
Government apps like CoronaApp				
Other				

11. How often do you browse social media?
    11.1. Less than 30 minutes
    11.2. Between 30 minutes to 2 hours
    11.3. Between 2 to 3 hours
    11.4. More than 3 hours

12. I searched for health-related information during the last year
    12.1. Yes
    12.2. No

13. I published health-related information during the last year
    13.1. Yes
    13.2. No

14. I published influenza vaccine-related information during the last year

	1-5 times	6-10 times	10 times and more	Not relevant or not familiar or not user
Twitter				
Facebook				
Tumblr				
Telegram				
Instagram				
Reddit				
Flickr				
Pinterest				
LinkedIn				

Government apps like CoronaApp				
Other				

15. To the best of my knowledge, the flu vaccine is
    15.1. An attenuated live virus
    15.2. Particulate vaccine

16. I got vaccinated against the flu in the last year (2019-2020)?
    16.1. Yes
    16.2. No

17. To the best of my knowledge, the influenza vaccine causes the influenza disease?
    17.1. Yes
    17.2. No

18. If you have been vaccinated against influenza in the past year (2019-2020), what is the reason for it?
    18.1. I saw advertisements in the media (newspapers / TV ..)
    18.2. I get vaccinated every year
    18.3. I got a reminder from my HMO
    18.4. I got information on Social Media
    18.5. I discussed with relatives (friends or family)
    18.6. I was not vaccinated
    18.7. Other

19. I searched for influenza vaccine-related information during the last year
    19.1. Yes
    19.2. No

20. I verify the information on social media with the attending physician/family physician?
    20.1. Yes
    20.2. Sometimes
    20.3. No

21. In case of conflict (difference of information content), do you think the information from the family physician is more reliable than the one you come across on social media?
    21.1. The physician's information is always more reliable than the one available on social media
    21.2. Often a physician's information is more reliable than the one available on social media
    21.3. The physician's information is rarely more reliable than the one available on social media
    21.4. Information on social media is always more reliable than the physician's opinion

22. Do you or a member of your family (up to the second degree) have a chronic disease (e.g. asthma, epilepsy, multiple sclerosis, hypertension, heart disease, diabetes, Crohn's, etc.)?
    22.1. Yes
    22.2. No

23. Please rate the level of information reliability about (influenza) vaccines on each of the following social media

	Not reliable	Sometimes reliable	Very reliable	Not relevant or not familiar or not user
Twitter				
Facebook				
Tumblr				
Telegram				
Instagram				
Reddit				
Flickr				
Pinterest				

LinkedIn				
Government apps like CoronaApp				
Other				

24. How do you determine that information about vaccines on social media is reliable? When the information is
    - 24.1. published by governmental and health organizations
    - 24.2. provided by a Healthcare professional
    - 24.3. Figuring in scientific publications
    - 24.4. Shared by someone I know
    - 24.5. Disseminated by the pharmaceutical industry
    - 24.6. Communicated by vaccine opponents
    - 24.7. From other sources

25. Please rate the influence of (influenza) vaccine information on each of the following social media

	Not influencing	Sometimes influencing	Very influencing	Not relevant or not familiar or not user
Twitter				
Facebook				
Tumblr				
Telegram				
Instagram				
Reddit				
Flickr				
Pinterest				
LinkedIn				
Government apps like CoronaApp				
Other				

26. I searched for SARS-CoV-2 / COVID19-related information during the last months
    26.1. Yes
    26.2. No

27. I published SARS-CoV-2 / COVID19-related information during the last months on the following social media

	1-5 times	6-10 times	10 times and more	Not relevant or not familiar or not user
Twitter				
Facebook				
Tumblr				
Telegram				
Instagram				
Reddit				
Flickr				
Pinterest				
LinkedIn				
Government apps like CoronaApp				
Other				

28. Please rate the level of information reliability about SARS-CoV-2 / COVID19 on each of the following social media

	Not reliable	Sometimes reliable	Very reliable	Not relevant or not familiar or not user
Twitter				

Facebook				
Tumblr				
Telegram				
Instagram				
Reddit				
Flickr				
Pinterest				
LinkedIn				
Government apps like CoronaApp				
Other				

29. How do you determine that information about SARS-CoV-2 / COVID19 on social media is reliable? When the information is
    - 29.1. published by governmental and health organizations
    - 29.2. provided by a Healthcare professional
    - 29.3. Figuring in scientific publications
    - 29.4. Shared by someone I know
    - 29.5. Disseminated by the pharmaceutical industry
    - 29.6. Communicated by vaccine opponents
    - 29.7. From other sources

30. Please rate the influence of SARS-CoV-2 / COVID19 information on the following social media

	Not influencing	Sometimes influencing	Very influencing	Not relevant or not familiar or not user
Twitter				
Facebook				
Tumblr				
Telegram				

Instagram				
Reddit				
Flickr				
Pinterest				
LinkedIn				
Government apps like CoronaApp				
Other				
