# Supplementary material for: Social Media Engagement and Influenza Vaccination During the COVID-19 Pandemic: Cross-sectional Survey Study
Source: J Med Internet Res. 2021 Mar 16;23(3):e25977. doi: 10.2196/25977 (PMC7968480; doi:10.2196/25977)
Supplement: Multimedia Appendix 2 [file jmir_v23i3e25977_app2.pdf]

		Vaccinated in 2019		
	**All participants**	**Yes**	**No**	**P value**
	N=207	***N=79***	***N=128***	
Daily time on social media				.536
Less than 30 minutes	43 (20.8%)	18 (22.8%)	25 (19.5%)	
Between 30 minutes and 2 hours	82 (39.6%)	31 (39.2%)	51 (39.8%)	
Between 2 and 3 hours	49 (23.7%)	15 (19.0%)	34 (26.6%)	
More than 3 hours	33 (15.9%)	15 (19.0%)	18 (14.1%)	
Facebook (Multifaceted Service)				.001
Active	76 (36.7%)	20 (25.3%)	56 (43.8%)	
Passive	39 (18.8%)	11 (13.9%)	28 (21.9%)	
No	92 (44.4%)	48 (60.8%)	44 (34.4%)	
Instagram (Photo and Video Sharing)				.001
Active	51 (24.6%)	13 (16.5%)	38 (29.7%)	
Passive	30 (14.5%)	5 (6.33%)	25 (19.5%)	
No	126 (60.9%)	61 (77.2%)	65 (50.8%)	
LinkedIn (Professional Networking)				.299
Active	21 (10.1%)	6 (7.59%)	15 (11.7%)	
Passive	39 (18.8%)	12 (15.2%)	27 (21.1%)	
No.	147 (71.0%)	61 (77.2%)	86 (67.2%)	
Telegram (Instant Messaging)				.014
Active	12 (5.80%)	4 (5.06%)	8 (6.25%)	
Passive	24 (11.6%)	3 (3.80%)	21 (16.4%)	
No	171 (82.6%)	72 (91.1%)	99 (77.3%)	
Others<br>(Twitter, Tumblr, Reddit, Flickr, Others)				.139
Active / Passive	38 (18.4%)	10 (12.7%)	28 (21.9%)	
No	169 (81.6%)	69 (87.3%)	100 (78.1%)	

Multimedia Appendix 2. Social media general usage and vaccination status against influenza in 2019
