# Supplementary material for: Social Media Engagement and Influenza Vaccination During the COVID-19 Pandemic: Cross-sectional Survey Study
Source: J Med Internet Res. 2021 Mar 16;23(3):e25977. doi: 10.2196/25977 (PMC7968480; doi:10.2196/25977)
Supplement: Multimedia Appendix 3 [file jmir_v23i3e25977_app3.pdf]

	All participants	Social Media User				Non-Social Media User			
		Social Media Users	Yearly vaccinated	Not yearly vaccinated	P value	No Social Media Users	Yearly vaccinated	Not yearly vaccinated	P value
	N=79	***N=31***	***N=14***	***N=17***		***N=48***	***N=23***	***N=25***	
I got a reminder from my HMO	18 (22.8%)	7 (22.6%)	1 (7.14%)	6 (35.3%)	.094	11 (22.9%)	2 (8.70%)	9 (36.0%)	.057
I discussed with relatives	14 (17.7%)	6 (19.4%)	3 (21.4%)	3 (17.6%)	1.000	8 (16.7%)	1 (4.35%)	7 (28.0%)	.050
I saw advertisement(s) on (social) media	9 (11.4%)	4 (12.9%)	0 (0.00%)	4 (23.5%)	.107	5 (10.4%)	0 (0.00%)	5 (20.0%)	.051
I got information on social media	5 (6.33%)	3 (9.68%)	2 (14.3%)	1 (5.88%)	.576	2 (4.17%)	1 (4.35%)	1 (4.00%)	1.000
Due to another reason	14 (17.7%)	6 (19.4%)	0 (0.00%)	6 (35.3%)	.021	8 (16.7%)	0 (0.00%)	8 (32.0%)	.004

Multimedia Appendix 3. Reasons for obtaining the influenza vaccine in 2019 (All participants and stratification by social media use and vaccination against influenza in 2019)
