# Supplementary material for: Social Media Engagement and Influenza Vaccination During the COVID-19 Pandemic: Cross-sectional Survey Study
Source: J Med Internet Res. 2021 Mar 16;23(3):e25977. doi: 10.2196/25977 (PMC7968480; doi:10.2196/25977)
Supplement: Multimedia Appendix 4 [file jmir_v23i3e25977_app4.pdf]

		Vaccinated in 2019		
	**All participants**	**Yes**	**No**	**P value**
	N=207	***N=79***	***N=128***	
I searched for health-related information				.627
Yes	128 (61.8%)	51 (64.6%)	77 (60.2%)	
No	79 (38.2%)	28 (35.4%)	51 (39.8%)	
I searched for influenza vaccine-related information				<.001
Yes	33 (15.9%)	22 (27.8%)	11 (8.59%)	
No	174 (84.1%)	57 (72.2%)	117 (91.4%)	
I published health-related information				.060
Yes	102 (49.3%)	46 (58.2%)	56 (43.8%)	
No	105 (50.7%)	33 (41.8%)	72 (56.2%)	
I published influenza vaccine-related information				.329
Yes	22 (10.6%)	11 (13.9%)	11 (8.59%)	
No	185 (89.4%)	68 (86.1%)	117 (91.4%)	
I searched for SARS-CoV-2 / COVID19-related information (including about a vaccine against it)				.022
Yes	49 (23.7%)	26 (32.9%)	23 (18.0%)	
No	158 (76.3%)	53 (67.1%	105 (82.0%)	
I published SARS-CoV-2 / COVID19-related information				1.00
Yes	12 (5.80%)	4 (5.06%)	8 (6.25%)	
No	195 (94.2%)	75 (94.9%)	120 (93.8%)	

Multimedia Appendix 4. Searching for and publishing information related to health and specifically to influenza vaccines and COVID-19 in the 12 months before participating in the survey
