# Supplementary material for: Social Media Engagement and Influenza Vaccination During the COVID-19 Pandemic: Cross-sectional Survey Study
Source: J Med Internet Res. 2021 Mar 16;23(3):e25977. doi: 10.2196/25977 (PMC7968480; doi:10.2196/25977)
Supplement: Multimedia Appendix 5 [file jmir_v23i3e25977_app5.pdf]

	Influenza- and vaccine-related information				SARS-CoV-2 / COVID-19- and vaccine-related information			
	**All participants**	**Yes**	**No**	**P value**	**All participants**	**Yes**	**No**	**P value**
	N=207	***N=79***	***N=128***		***N=207***	***N=79***	***N=128***	
Reliability of Facebook				.056				.003
Yes	71 (34.3%)	21 (26.6%)	50 (39.1%)		74 (35.7%)	21 (26.6%)	53 (41.4%)	
No	16 (7.73%)	4 (5.06%)	12 (9.38%)		19 (9.18%)	3 (3.80%)	16 (12.5%)	
No opinion	120 (58.0%)	54 (68.4%)	66 (51.6%)		114 (55.1%)	55 (69.6%)	59 (46.1%)	
Influenced by Facebook				.016				.019
Yes	36 (17.4%)	10 (12.7%)	26 (20.3%)		62 (30.0%)	19 (24.1%)	43 (33.6%)	
No	53 (25.6%)	14 (17.7%)	39 (30.5%)		28 (13.5%)	6 (7.59%)	22 (17.2%)	
No opinion	118 (57.0%)	55 (69.6%)	63 (49.2%)		117 (56.5%)	54 (68.4%)	63 (49.2%)	
Reliability of Instagram				.040				.004
Yes	22 (10.6%)	4 (5.06%)	18 (14.1%)		27 (13.0%)	6 (7.59%)	21 (16.4%)	
No	20 (9.66%)	5 (6.33%)	15 (11.7%)		15 (7.25%)	1 (1.27%)	14 (10.9%)	
No opinion	165 (79.7%)	70 (88.6%)	95 (74.2%)		165 (79.7%)	72 (91.1%)	93 (72.7%)	
Influenced by Instagram				.033				.017
Yes	10 (4.83%)	3 (3.80%)	7 (5.47%)		30 (14.5%)	7 (8.86%)	23 (18.0%)	
No	42 (20.3%)	9 (11.4%)	33 (25.8%)		18 (8.70%)	3 (3.80%)	15 (11.7%)	
No opinion	155 (74.9%)	67 (84.8%)	88 (68.8%)		159 (76.8%)	69 (87.3%)	90 (70.3%)	
Reliability of LinkedIn				.569				.014
Yes	21 (10.1%)	6 (7.59%)	15 (11.7%)		22 (10.6%)	4 (5.06%)	18 (14.1%)	
No	13 (6.28%)	4 (5.06%)	9 (7.03%)		10 (4.83%)	1 (1.27%)	9 (7.03%)	
No opinion	173 (83.6%)	69 (87.3%)	104 (81.2%)		175 (84.5%)	74 (93.7%)	101 (78.9%)	
Influenced by LinkedIn				.013				.131
Yes	5 (2.42%)	0 (0.00%)	5 (3.91%)		13 (6.28%)	2 (2.53%)	11 (8.59%)	
No	39 (18.8%)	9 (11.4%)	30 (23.4%)		26 (12.6%)	8 (10.1%)	18 (14.1%)	
No opinion	163 (78.7%)	70 (88.6%)	93 (72.7%)		168 (81.2%)	69 (87.3%)	99 (77.3%)	
Reliability of Telegram				.147				.017
Yes	15 (7.25%)	3 (3.80%)	12 (9.38%)		18 (8.70%)	3 (3.80%)	15 (11.7%)	
No	16 (7.73%)	4 (5.06%)	12 (9.38%)		14 (6.76%)	2 (2.53%)	12 (9.38%)	
No opinion	176 (85.0%)	72 (91.1%)	104 (81.2%)		175 (84.5%)	74 (93.7%)	101 (78.9%)	

Influenced by Telegram				.018				.070
Yes	6 (2.90%)	0 (0.00%)	6 (4.69%)		13 (6.28%)	2 (2.53%)	11 (8.59%)	
No	33 (15.9%)	8 (10.1%)	25 (19.5%)		24 (11.6%)	6 (7.59%)	18 (14.1%)	
No opinion	168 (81.2%)	71 (89.9%)	97 (75.8%)		170 (82.1%)	71 (89.9%)	99 (77.3%)	
Reliability of other SNSs				.911				.278
Yes	5 (2.42%)	2 (2.53%)	3 (2.34%)		5 (2.42%)	1 (1.27%)	4 (3.12%)	
No	10 (4.83%)	3 (3.80%)	7 (5.47%)		8 (3.86%)	1 (1.27%)	7 (5.47%)	
No opinion	192 (92.8%)	74 (93.7%)	118 (92.2%)		194 (93.7%)	77 (97.5%)	117 (91.4%)	
Influenced by other SNSs				.360				.554
Yes					6 (2.90%)	3 (3.80%)	3 (2.34%)	
No	28 (13.5%)	8 (10.1%)	20 (15.6%)		18 (8.70%)	5 (6.33%)	13 (10.2%)	
No opinion	179 (86.5%)	71 (89.9%)	108 (84.4%)		183 (88.4%)	71 (89.9%)	112 (87.5%)	

Multimedia Appendix 5. Perception of reliability and influence of the information related to “influenza and vaccine” and COVID-19 available on the most used social media platforms
