# Supplementary material for: Social Media Engagement and Influenza Vaccination During the COVID-19 Pandemic: Cross-sectional Survey Study
Source: J Med Internet Res. 2021 Mar 16;23(3):e25977. doi: 10.2196/25977 (PMC7968480; doi:10.2196/25977)
Supplement: Multimedia Appendix 6 [file jmir_v23i3e25977_app6.pdf]

		Social Media User		
	**All participants**	**Yes**	**No**	**P value**
	N=207	*N=119*	*N=88*	
Reliability of Facebook				<.001
Yes	71 (34.3%)	70 (58.8%)	1 (1.14%)	
No	16 (7.73%)	16 (13.4%)	0 (0.00%)	
No opinion	120 (58.0%)	33 (27.7%)	87 (98.9%)	
Influenced by Facebook:				<.001
Yes	36 (17.4%)	36 (30.3%)	0 (0.00%)	
No	53 (25.6%)	53 (44.5%)	0 (0.00%)	
No opinion	118 (57.0%)	30 (25.2%)	88 (100%)	
Reliability on Instagram:				<.001
Yes	22 (10.6%)	22 (18.5%)	0 (0.00%)	
No	20 (9.66%)	19 (16.0%)	1 (1.14%)	
No opinion	165 (79.7%)	78 (65.5%)	87 (98.9%)	
Influenced by Instagram:				<.001
Yes	10 (4.83%)	10 (8.40%)	0 (0.00%)	
No	42 (20.3%)	42 (35.3%)	0 (0.00%)	
No opinion	155 (74.9%)	67 (56.3%)	88 (100%)	
Reliability of LinkedIn				<.001
Yes	21 (10.1%)	20 (16.8%)	1 (1.14%)	
No	13 (6.28%)	13 (10.9%)	0 (0.00%)	
No opinion	173 (83.6%)	86 (72.3%)	87 (98.9%)	
Influenced by Linkedin				<.001
Yes	5 (2.42%)	5 (4.20%)	0 (0.00%)	
No	39 (18.8%)	39 (32.8%)	0 (0.00%)	
No opinion	163 (78.7%)	75 (63.0%)	88 (100%)	
Reliability of Telegram				<.001
Yes	15 (7.25%)	14 (11.8%)	1 (1.14%)	
No	16 (7.73%)	16 (13.4%)	0 (0.00%)	
No opinion	176 (85.0%)	89 (74.8%)	87 (98.9%)	
Influenced by Telegram				<.001
Yes	6 (2.90%)	6 (5.04%)	0 (0.00%)	
No	33 (15.9%)	33 (27.7%)	0 (0.00%)	
No opinion	168 (81.2%)	80 (67.2%)	88 (100%)	

Reliability of other SNSs				0.001
Yes	5 (2.42%)	5 (4.20%)	0 (0.00%)	
No	10 (4.83%)	10 (8.40%)	0 (0.00%)	
No opinion	192 (92.8%)	104 (87.4%)	88 (100%)	
Influenced by other SNSs				<0.001
Yes	0(0.00%)	0(0.00%)	0(0.00%)	
No	28 (13.5%)	28 (23.5%)	0 (0.00%)	
No opinion	179 (86.5%)	91 (76.5%)	88 (100%)	

Multimedia Appendix 6. Perception of reliability and influence of the information related to "influenza and vaccine" and COVID-19 available on the most used social media platforms between social media users and non-social media users
