# Supplementary material for: Social Media Engagement and Influenza Vaccination During the COVID-19 Pandemic: Cross-sectional Survey Study
Source: J Med Internet Res. 2021 Mar 16;23(3):e25977. doi: 10.2196/25977 (PMC7968480; doi:10.2196/25977)
Supplement: Multimedia Appendix 7 [file jmir_v23i3e25977_app7.pdf]

| | | Vaccinated in 2019 | | |
|---|---|---|---|---|
| | **All participants** | **Yes** | **No** | **P value** |
| | **N=207** | **N=79** | **N=128** | |
| Governmental and health organizations | | | | .078 |
| Yes | 155 (74.9%) | 65 (82.3%) | 90 (70.3%) | |
| No | 52 (25.1%) | 14 (17.7%) | 38 (29.7%) | |
| Healthcare professional | | | | .061 |
| Yes | 84 (40.6%) | 39 (49.4%) | 45 (35.2%) | |
| No | 123 (59.4%) | 40 (50.6%) | 83 (64.8%) | |
| Scientific publications | | | | 1.00 |
| Yes | 70 (33.8%) | 27 (34.2%) | 43 (33.6%) | |
| No | 137 (66.2%) | 52 (65.8%) | 85 (66.4%) | |
| Someone I know | | | | 1.00 |
| Yes | 17 (8.21%) | 6 (7.59%) | 11 (8.59%) | |
| No | 190 (91.8%) | 73 (92.4%) | 117 (91.4%) | |
| Pharmaceutical industry | | | | 1.00 |
| Yes | 3 (1.45%) | 1 (1.27%) | 2 (1.56%) | |
| No | 204 (98.6%) | 78 (98.7%) | 126 (98.4%) | |
| Vaccine opponents | | | | .651 |
| Yes | 5 (2.42%) | 1 (1.27%) | 4 (3.12%) | |
| No | 202 (97.6%) | 78 (98.7%) | 124 (96.9%) | |
| Other sources | | | | .229 |
| Yes | 18 (8.70%) | 4 (5.06%) | 14 (10.9%) | |
| No | 189 (91.3%) | 75 (94.9%) | 114 (89.1%) | |

**Multimedia Appendix 7.** Confidence in sources of information about vaccine against influenza
