# Supplementary material for: Social Media Engagement and Influenza Vaccination During the COVID-19 Pandemic: Cross-sectional Survey Study
Source: J Med Internet Res. 2021 Mar 16;23(3):e25977. doi: 10.2196/25977 (PMC7968480; doi:10.2196/25977)
Supplement: Multimedia Appendix 8 [file jmir_v23i3e25977_app8.pdf]

Dataset	Tree identifier	Prediction Performance (in %)
Socio-demographics (reported in table 1)	1	59.1%
Social media general usage (reported in table 2)	2	56.3%
Reasons for obtaining the influenza vaccine in 2019 (reported in table 3)	3	80.3%
Searching and publishing information (reported in table 4)	4	64.7%
Perception of reliability and influence of the mainly used social media platforms for “Influenza and vaccines” related information (reported in table 5)	5	56.3%
Perception of reliability and influence of the most used social media platforms for COVID-19 related information (reported in table 5)	6	56.3%
Confidence in sources of information (reported in table 6)	7	54.9%
Overall attributes (questions) of the survey	8	77.5%
Overall attributes with $P \leq .10$	9	80.3%
Overall attributes with $P \leq .10$ and without COVID-19 related attributes		
Overall attributes with $P \leq .10$ and without “due to another reason” (reasons for being vaccinated)	10	76,1%
Overall attributes with $P \leq .10$ and without “due to another reason” (reasons for being vaccinated) and without COVID-19 related data		

Multimedia Appendix 8. Prediction capabilities of decision trees based on the survey data
